# Associations between outdoor air pollutants and non-viral asthma exacerbations and airway inflammatory responses in children and adolescents living in urban areas in the USA: a retrospective secondary analysis

**DOI:** 10.1016/S2542-5196(22)00302-3

**Published:** 2023-01

**Authors:** Matthew C Altman, Meyer Kattan, George T O’Connor, Ryan C Murphy, Elizabeth Whalen, Petra LeBeau, Agustin Calatroni, Michelle A Gill, Rebecca S Gruchalla, Andrew H Liu, Stephanie Lovinsky-Desir, Jacqueline A Pongracic, Carolyn M Kercsmar, Gurjit K Hershey Khurana, Edward M Zoratti, Stephen J Teach, Leonard B Bacharier, Lisa M Wheatley, Steve M Sigelman, Peter J Gergen, Alkis Togias, William W Busse, James E Gern, Daniel J Jackson

**Affiliations:** Department of Medicine, University of Washington, Seattle, WA, USA (M C Altman MD, R C Murphy MD); Systems Immunology Division, Benaroya Research Institute, Seattle, WA, USA (M C Altman, E Whalen PhD); Columbia University, New York, NY, USA (Prof M Kattan MD, S Lovinsky-Desir MD); Department of Medicine, Boston University School of Medicine, Boston University, Boston, MA, USA (Prof G T O’Connor MD); Rho, Chapel Hill, NC, USA (P LeBeau PhD, A Calatroni MS); UT Southwestern Medical Center, Dallas, TX, USA (Prof M A Gill MD PhD, Prof R S Gruchalla MD PhD); Children’s Hospital Colorado, University of Colorado School of Medicine, Aurora, CO, USA (Prof A H Liu MD); Ann Robert H Lurie Children’s Hospital of Chicago, Chicago, IL, USA (Prof J A Pongracic MD); Cincinnati Children’s Hospital, Cincinnati, OH, USA (C M Kercsmar MD, Prof G K Khurana Hershey MD PhD); Henry Ford Health System, Detroit, MI, USA (Prof E M Zoratti MD); Children’s National Hospital, Washington, DC, USA (Prof S J Teach MD); Division of Allergy, Immunology, and Pulmonary Medicine, Washington University, Saint Louis, MO, USA (Prof L B Bacharier MD); NIH/NIAID, Bethesda, MD, USA (L M Wheatley MD, S M Sigelman RN, P J Gergen MD, A Togias MD); University of Wisconsin School of Medicine and Public Health, University of Wisconsin, Madison, WI, USA(Prof W W Busse MD, Prof J E Gern MD, Prof D J Jackson MD) Correspondence to: Dr Matthew C Altman, Systems Immunology Division, Benaroya Research Institute, Seattle, WA 98101, USA

## Abstract

**Background:**

Asthma prevalence and severity have markedly increased with urbanisation, and children in low-income urban centres have among the greatest asthma morbidity. Outdoor air pollution has been associated with adverse respiratory effects in children with asthma. However, the mechanisms by which air pollution exposure exacerbates asthma, and how these mechanisms compare with exacerbations induced by respiratory viruses, are poorly understood. We aimed to investigate the associations between regional air pollutant concentrations, respiratory illnesses, lung function, and upper airway transcriptional signatures in children with asthma, with particular focus on asthma exacerbations occurring in the absence of respiratory virus.

**Methods:**

We performed a retrospective analysis of data from the MUPPITS1 cohort and validated our findings in the ICATA cohort. The MUPPITS1 cohort recruited 208 children aged 6–17 years living in urban areas across nine US cities with exacerbation-prone asthma between Oct 7, 2015, and Oct 18, 2016, and monitored them during reported respiratory illnesses. The last MUPPITS1 study visit occurred on Jan 6, 2017. The ICATA cohort recruited 419 participants aged 6–20 years with persistent allergic asthma living in urban sites across eight US cities between Oct 23, 2006, and March 25, 2008, and the last study visit occurred on Dec 30, 2009. We included participants from the MUPPITS1 cohort who reported a respiratory illness at some point during the follow-up and participants from the ICATA cohort who had nasal samples collected during respiratory illness or at a scheduled visit. We used air quality index values and air pollutant concentrations for PM_2·5_, PM_10_, O_3_, NO_2_, SO_2_, CO, and Pb from the US Environmental Protection Agency spanning the years of both cohorts, and matched values and concentrations to each illness for each participant. We investigated the associations between regional air pollutant concentrations and respiratory illnesses and asthma exacerbations, pulmonary function, and upper airway transcriptional signatures by use of a combination of generalised additive models, case crossover analyses, and generalised linear mixed-effects models.

**Findings:**

Of the 208 participants from the MUPPITS1 cohort and 419 participants from the ICATA cohort, 168 participants in the MUPPITS1 cohort (98 male participants and 70 female participants) and 189 participants in the ICATA cohort (115 male participants and 74 female participants) were included in our analysis. We identified that increased air quality index values, driven predominantly by increased PM_2·5_ and O_3_ concentrations, were significantly associated with asthma exacerbations and decreases in pulmonary function that occurred in the absence of a provoking viral infection. Moreover, individual pollutants were significantly associated with altered gene expression in coordinated inflammatory pathways, including PM_2·5_ with increased epithelial induction of tissue kallikreins, mucus hypersecretion, and barrier functions and O_3_ with increased type-2 inflammation.

**Interpretation:**

Our findings suggest that air pollution is an important independent risk factor for asthma exacerbations in children living in urban areas and is potentially linked to exacerbations through specific inflammatory pathways in the airway. Further investigation of these potential mechanistic pathways could inform asthma prevention and management approaches.

**Funding:**

National Institutes of Health, National Institute of Allergy and Infectious Diseases.

## Introduction

Outdoor air pollution has been linked to the onset^1–3^ and exacerbation^4–7^ of asthma, with many pollutants associated with asthma exacerbations, most notably fine particulate matter (PM_2·5_),^8,9^ oxidising gases, including O_3_^10,11^ and NO_2_,^12,13^ and reductants, such as SO_2_.^14^ The negative effects of air pollution are likely to be particularly relevant in urban centres, where pollution exposure is high and the prevalence and morbidity of asthma in children is disproportionately high compared with rural and less dense urban areas.^15^ However, although physiological effects of air pollutants have been investigated, the molecular mechanisms by which air pollutants trigger asthma exacerbations are poorly understood, especially in susceptible populations, such as children with severe or uncontrolled asthma.^16^ Studies of the molecular effects of air pollutants during respiratory illnesses have not been conducted in humans.

We aimed to improve characterisation of the relationship between outdoor air pollution and asthma exacerbations among children living in urban settings by analysing changes in airway physiology and airway gene expression during acute exacerbations and then identifying associations between overall air quality and individual pollutants with distinct gene expression pathways. We performed a secondary analysis of the Mechanisms Underlying Asthma Exacerbations Prevented and Persistent with Immune-based Therapy Part 1 (MUPPITS1) cohort,^17^ integrating data for air quality to investigate the contribution of air pollution to molecular and physiological mechanisms of exacerbation pathogenesis. We then validated key observations from this cohort in the Inner-City Anti-IgE Therapy for Asthma (ICATA) cohort.^18^

## Methods

### Study design and participants

We performed a retrospective analysis of data from a prospective observational cohort study (MUPPITS1)^17^ and validated our findings with data from a randomised, double-blind, placebo-controlled trial in an independent cohort (ICATA).^18^

The MUPPITS1 study was a longitudinal observational study of 208 children with exacerbation-prone asthma in low-income urban centres, who were followed up between 2015 and 2017 for respiratory illnesses. This study has previously been described elsewhere.^17^ Briefly, participants were recruited between Oct 7, 2015, and Oct 18, 2016, across hospital clinics in major urban areas in nine US cities. An individual was eligible for enrolment if they were aged 6–17 years; were diagnosed with asthma by a clinician more than 1 year before recruitment; had at least two asthma exacerbations (ie, required systemic corti costeroids or hospital admission, or both) in the year before recruitment; were treated with at least one puff of fluticasone 250 μg twice daily, or its equivalent for children aged 6–11 years, or treated with at least one puff of fluticasone 250 μg plus salmeterol 50 μg twice daily, or its equivalent for children aged 12 years and older; had more than or equal to 150 peripheral blood eosinophils per mm^3^; did not smoke; and lived in a census tract with a density of more than or equal to 1000 families per square mile and with at least 10% of families with income below the poverty level (based on American Community Survey data^19^). Participants were identified for recruitment through the Registry for Asthma Characterization and Recruitment 2 and site-approved recruitment sources (NCT02513264). Participants were followed up prospectively for up to two respiratory illnesses or approximately 6 months, whichever occurred first. Participants who reported a respiratory illness were asked to return to the clinic twice in the 6-day period after the start of symptoms for collection of nasal samples and pulmonary function testing. Each illness was defined as a viral (V+) or non-viral (V–) event on the basis of virological assessment of the first nasal blow sample by use of the Luminex Respiratory Viral Panel (Luminex, Austin, TX, USA), with (Ex+) or without an asthma exacerbation (Ex–) on the basis of whether the participant was treated with systemic corticosteroids within 10 days following the onset of the respiratory event or not (appendix p 11). The last study visit occurred on Jan 6, 2017.

The ICATA study was a randomised, double-blind, placebo-controlled trial of omalizumab conducted from 2006 to 2009 in 419 inner-city children, adolescents, and young adults (ie, aged 6–20 years) with persistent allergic asthma, as previously described.^18^ Briefly, participants were enrolled between Oct 23, 2006, and March 25, 2008, across hospital clinics in major urban areas in eight US cities. An individual was eligible for enrolment if they were aged 6–20 years; were diagnosed with asthma by a clinician more than a year before recruitment or diagnosed with asthma and had symptoms for longer than 1 year; had bodyweight and total serum IgE suitable for omalizumab dosing and a positive skin-prick test to at least one perennial allergen; did not smoke; and lived in a census tract with a density of more than or equal to 1000 families per square mile with and at least 10% of families with income below the poverty level. In an exploratory substudy of 189 of 419 participants in four of eight US cities (ie, New York, NY, Chicago, IL, Dallas, TX, and Cleveland, OH), 100 nasal samples were collected within 7 days of the onset of an asthma exacerbation (ie, required systemic corticosteroids; Ex+) and 165 nasal samples were collected at study week 48 in the absence of an exacerbation. The last study visit occurred on Dec 30, 2009. The nasal samples were used for virological assessment by use of the Eragen Multi-Code Respiratory Virus Panel (Eragen Biosciences, Madison, WI, USA) and defined as V+ or V–(appendix pp 12–13).

In this analysis, we included participants from the MUPPITS1 cohort who reported a respiratory event at some point during the follow-up and participants from the ICATA cohort who had nasal samples collected due to a respiratory event or at a scheduled visit. Sex was self-reported by participants, with the options of male or female.

The MUPPITS1 and ICATA protocols were approved by the Inner-City Asthma Consortium steering committee, protocol review committee, and data safety monitoring board. The MUPPITS1 protocol^20^ was reviewed by a single institutional review board, and the ICATA protocol^21^ was reviewed by the institutional review boards of all participating institutions. Written informed consent for the MUPPITS1 and ICATA studies was obtained from the parents or legal guardians of all participants and applies to this analysis.

### Procedures

To quantify exposure to air pollution, we downloaded air quality index (AQI) values and individual air pollutant data for PM_2·5_, PM_10_, O_3_, NO_2_, SO_2_, CO, and Pb from the US Environmental Protection Agency (EPA),^22^ spanning the years of the studies (2015–17 for MUPPITS1 and 2006–09 for ICATA) and the cities of participant recruitment. The EPA uses set formulas to convert raw measurements of pollutants into a summary AQI (on the basis of the single pollutant with the most hazardous concentration on a given day) for each day and each core-based statistical area (ie, geographical area anchored by an urban centre of at least 10 000 people plus adjacent areas with socioeconomic ties).^23^ AQI data were matched to each event for each participant according to the corebased statistical area in which they lived and the date in relation to their reported respiratory illness symptoms. For each pollutant, monitors within each core-based statistical area were subset to include only those in the recruitment census tract, excluding monitors that were in the core-based statistical area but outside of the census tract. For each pollutant, the maximum concentration was then taken for each city census tract and day and similarly matched to each event according to core-based statistical area and date.

In the MUPPITS1 cohort, a subset of respiratory events that were recorded had detailed assessment available, including virological and transcriptomic assessments, pulmonary function tests, and cell differentials, from serial nasal blow and lavage samples collected after the reported onset of respiratory illness symptoms, as previously described.^17^ Briefly, viral status was established on the basis of the results of multiplex PCR (Luminex Respiratory Viral Panel, Luminex, Austin, TX, USA) and partial sequencing of nasal blow samples to identify respiratory virus species; cell differentials were determined by cytospin of nasal lavage samples quantify ing neutrophils, lymphocytes, macrophages, eosinophils, respiratory epithelium, and squamous cells; and RNA-sequencing was performed on bulk RNA extracted from nasal lavage cell pellets and is publicly available at Gene Expression Omnibus, accession number GSE115824. RNA-sequencing data were summarised into cell-associated modules of coexpressed genes by use of combined cell association by correlation and weighted gene correlation network analysis^24^ and annotated by use of Database for Annotation, Visualization and Integrated Discovery^25^ and Search Tool for the Retrieval of Interacting Genes/Proteins;^26^ the modules used in this analysis have been previously described.^17^ Spirometry and the measurement of fractional exhaled NO (FeNO) were collected according to American Thoracic Society and European Respiratory Society guidelines.^27^ In the ICATA cohort, nasal-secretion samples were collected at the onset of an asthma exacerbation or at a routine study visit 48 weeks after study enrolment, and RNA was extracted and analysed for respiratory viruses by the Eragen Multi-Code Respiratory Virus Panel.^18^

### Outcomes

The key outcome was association between AQI values and pollutant concentrations and the respiratory event type. Other outcomes included associations with pulmonary function and nasal module expression values. All outcomes were modelled in relation to the AQI value and concentrations of pollutants.

### Statistical analysis

Differences in AQI and air pollution in MUPPITS1 were assessed by comparing mean values of each metric among specified event groups (ie, among the four event subgroups [V–Ex+, V+Ex+, V–Ex–, and V+Ex–] or between event groups [ie, Ex+ and Ex–]) across timespans before and after the onset of respiratory illness symptoms, designated day 0, and during the onset of asthma exacerbations. In ICATA, differences were compared among groups (V–Ex+, V+Ex+, V– at week 48, and V+ at week 48) relative to the day of nasal sampling, designated day 0. Longitudinal differences among groups were visualised and statistically compared among the groups by generalised additive models,^28–30^ including the day relative to respiratory illness symptom onset as the smoothed, continuous variable, and a term for event group. Covariates were added for city of residence or season, where specified. The generalised additive model tested whether the four (or two) groups were different over the entire timespan included in the model. For the four-group comparisons, ANOVA was run to establish whether the four groups were different by use of the Wald test in the generalised additive model, followed by post-hoc pairwise comparisons. The same model syntax and data visualisation were used for both MUPPITS1 and ICATA. Responsible pollutants for AQI values for each day were those defined by the EPA. Case crossover analyses of Ex+ events in MUPPITS1 were performed comparing days –7 to +1 (case) relative to the date of exacerbation onset (designated day 0) to days –16 to –8 (control) or days –37 to –8 (control) as specified. Longitudinal differences were visualised by generalised additive models, and statistical comparisons between time periods were tested by conditional logistic regression,^31^ including a covariate for day of the week. Lung function measurements (forced expiratory volume in 1 s as a percentage of predicted [FEV_1_% predicted] and ratio of forced expiratory volume in 1 s to forced vital capacity [FEV_1_/FVC]) were compared with AQI values or pollutant concentrations from the same day by use of generalised linear mixed-effects models split by event subgroup with a random effect for participant to account for the correlation between values from the same participants; values collected after the initiation of systemic corticosteroids were excluded for this analysis, and for FEV_1_/FVC the model also controlled for age and sex. After testing AQI and pollutants, p values were adjusted with the Benjamini-Hochberg method to identify those with a false discovery rate (FDR) of less than 0·05. Module expression was compared with AQI values or pollutant concentrations summed over 3 days (ie, the day of sample collection and the preceding 2 days) by use of a generalised linear mixed-effects model adjusted for cell percentages in the nasal lavage sample and the number of counts in the RNA-sequencing library with a random effect for participant to account for the correlation between values from the same participants; samples collected after the initiation of systemic corticosteroids were excluded for this analysis. Multiple-testing correction for module analysis was performed with the Benjamini-Hochberg method and modules with FDR less than 0·05 were considered significant. Demographic and clinical characteristic variables among event or sample subgroups in each cohort were summarised with median and IQR for continuous variables and count and percentage for categorical variables. Within each cohort, V+ and V– events or samples were compared by use of generalised linear mixed-effect models, assuming a binomial distribution for the categorical variables and ranked continuous variables, and included a random effect for participant to account for the correlation between values from the same participants. In cases where at least one of the categories of a categorical variable was zero, a Fisher’s exact test was used instead.

Wherever data were missing they were excluded from the analysis. The number of samples for each analysis are indicated in the figure legends. Every reported respiratory event in MUPPITS1 and every timepoint with a nasal sample in ICATA were included. For all analyses we used R (version 4.1.0) and all figures were constructed with R package ggplot2 (version 3.3.6).

### Role of the funding source

National Institute of Allergy and Infectious Disease project scientists had no role in study design or data analysis but participated collaboratively in the interpretation and writing of the report.

## Results

Of the 208 participants from the MUPPITS1 cohort and 419 participants from the ICATA cohort, 168 participants in the MUPPITS1 cohort (98 male participants and 70 female participants) and 189 participants in the ICATA cohort (115 male participants and 74 female participants) were included in our analysis.

336 respiratory events were recorded in the MUPPITS1 cohort, of which 143 resulted in asthma exacerbations (Ex+) and 193 did not (Ex–). AQI values were significantly higher (ie, indicating worsened air quality) for the Ex+ events compared with Ex– events across the duration of 9 days before the reported start of respiratory symptoms to 9 days after (p<0·0001; appendix p 2). Among these events, 154 had detailed assessments during the event and could, therefore, be subdivided into either V+ or V– events. Notably, V– events accounted for 52 (33·8%) of 154 events, and V–Ex+ events accounted for 14 (29·8%) of 47 of Ex+ events (table 1, appendix p 11).

**Figure 1:**
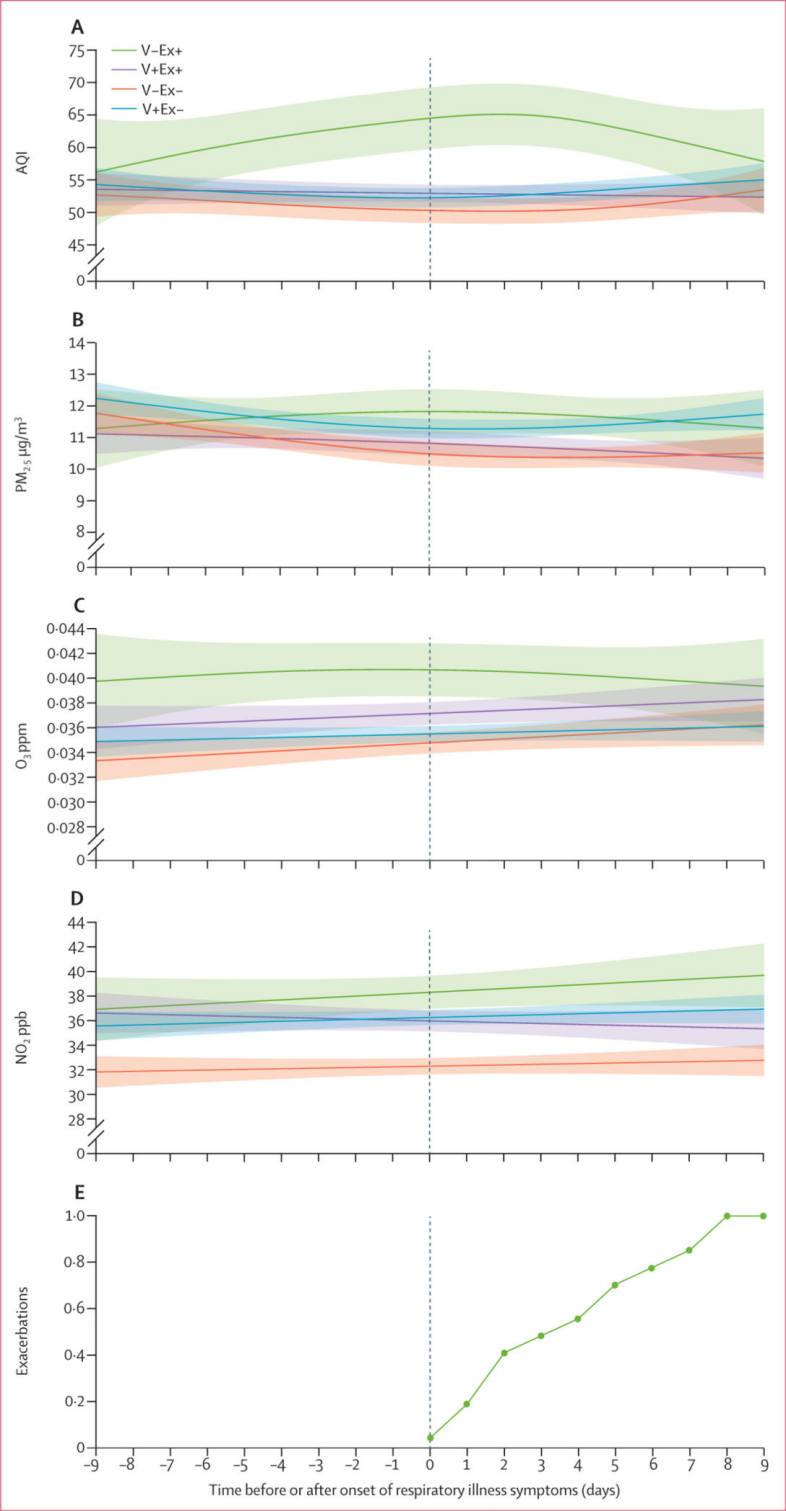
AQI values and individual pollutant concentrations over time Trend over 9 days before the reported start of respiratory illness symptoms (day 0) to 9 days after in AQI (A), PM_2·5_ concentrations (B), O_3_ concentrations (C), NO_2_ concentrations (D), and cumulative incidence of exacerbations in the V–Ex+ subgroup, where 1·0 represents 14 exacerbations (E). Data were plotted using generalised additive model fits showing 95% CIs. There were 14 events in the V–Ex+ group, 33 events in the V+Ex+ group, 38 events in the V–Ex– group, and 69 events in the V+Ex– group. Equivalent plots showing all data points are shown in the appendix (p 3). AQI=air quality index. V–Ex+=non-viral event with exacerbation. V+Ex+=viral event with exacerbation. V–Ex–=non-viral event without exacerbation. V+Ex–=viral event without exacerbation.

AQI values were specifically increased in the V–Ex+ event subgroup, compared with the other three event subgroups across the duration of 9 days before the reported start of respiratory symptoms to 9 days after the start (p<0·0001; figure 1A). The AQI values appeared to increase during the period of days around clinical asthma exacerbations in the V–Ex+ group, and exacerbations occurred after several days of mean sustained increase in AQI (figure 1A, 1E; appendix p 3). The other three groups had statistically similar AQI values to one another. V–Ex+ events were thereby largely responsible for the higher AQI values among Ex+ events than among Ex– events (appendix p 2). Similarly, when comparing AQI values over the 9 days before exacerbation onset, V–Ex+ events had sustained higher AQI values than did V+Ex+ events (p<0·0001). We performed a case-crossover comparison that also showed significantly higher AQI values in the V–Ex+ event subgroup in the week immediately before exacerbation onset (ie, days –7 to +1) than in the week before this period (ie, days –16 to –8; p=0·0075; appendix p 4), or in the 30 days before this period (ie, days –37 to –8; p=0·016), which was not true for the V+Ex+ event subgroup.

The EPA-defined pollutants used to calculate AQI values during V–Ex+ events were O_3_ (136 [51·1%] of 266 total days analysed), NO_2_ (47 [17·7%] days), and PM_2·5_ (82 [30·8%] days) but not PM_10_ (1 [0·4%] day), CO (0 days), or SO_2_ (0 days). Correspondingly, the concentrations of each of these three pollutants were significantly higher in the V–Ex+ group than in the other three groups during this same window of time (figure 1B–1D; appendix p 3; O_3_ p<0·0001, NO_2_ p<0·0001, PM_2·5_ p=0·0006). The V+Ex+ group also had modestly higher O_3_ concentrations than the two Ex– groups (p=0·0014; figure 1C; appendix p 3). Case-crossover analyses supported similar trends for these three pollutants (appendix p 4). Mean PM_2·5_ concentrations during the V–Ex+ events on some occasions reached concentrations higher than the EPA primary standards (ie, 12·0 μg/m^3^ annual mean), but mean O_3_ and NO_2_ concentrations did not exceed US National Ambient Air Quality Standards.^32^ Similarly, mean AQI assessments did not reach EPA concentrations designated as unhealthy (defined as AQI of 151–200) or unhealthy for sensitive groups (defined as 101–150 for people with heart and lung disease, older adults, children, people with diabetes, and people of lower socioeconomic status) but rather were at concentrations in the moderate category (defined as 51–100 AQI) for several days.^33^

V–Ex+ events were identified in only five of the nine cities with participants in the study. Adjusting the primary model for city showed similar results for AQI (ie, AQI values were higher for the duration of the 9 days before the reported start of respiratory symptoms to 9 days after in the V–Ex+ group), as did a subgroup analysis restricted to individuals living in only those five cities (p<0·0001; appendix p 5). V–Ex+ events occurred throughout the year, although were numerically but not significantly more likely than V+Ex+ events to occur in the summer. Adjusting the primary model for season showed similar results for AQI (data not shown; p<0·0001). Subsetting by season showed higher AQI associated with V–Ex+ events than with the other types of event both in spring and summer (p<0·0001) and autumn and winter (p=0·0003), although the magnitude of difference was most pronounced in spring and summer (appendix p 6).

**Figure 2:**
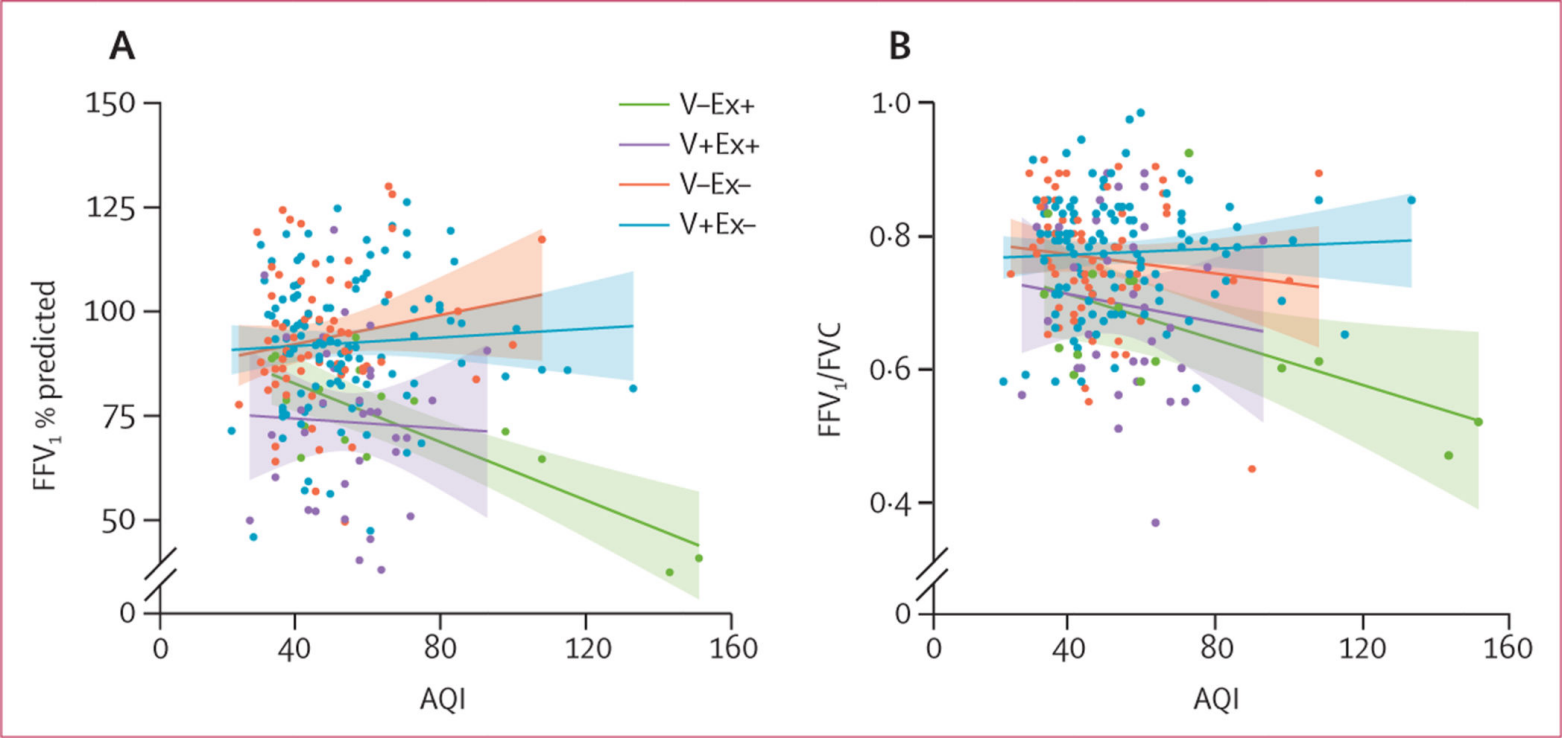
Associations of pulmonary functions with AQI values Associations of FEV_1_% predicted (A) and FEV_1_/FVC ratio (B) with AQI measured on the same day. Regression lines, 95% CIs, and all data points are shown for each group. There were 16 data points for the V–Ex+ group, 36 data points for the V+Ex+ group, 70 data points for the V–Ex– group, and 116 data points for the V+Ex– group. AQI=air quality index. FEV_1_% predicted=forced expiratory volume in 1 s as a percentage of predicted. FEV_1_/FVC=ratio of forced expiratory volume in 1 s to forced vital capacity. V–Ex+=non-viral event without exacerbation. V+Ex+=viral event with exacerbation. V–Ex–=non-viral event without exacerbation. V+Ex–=viral event without exacerbation.

The V–Ex+ event subgroup had physiological airway obstruction, as measured by FEV_1_% predicted, that was not significantly different to the V+Ex+ subgroup (p=0·94) and similar FeNO (table 1; p=0·66). There were no significant differences in urine cotinine concentrations, as a measure of tobacco smoke exposure, between groups (table 1). During V–Ex+ events, AQI values showed a significant inverse association with FEV_1_% predicted (linear mixed effect model parameter estimate [β]=–0·35, FDR=0·014) and FEV_1_/FVC ratio (β=–0·0028, FDR=0·014; figure 2). No significant inverse association was identified in the other three groups. Among the individual pollutants, O_3_ was significantly associated with FEV_1_% predicted (β=–591, FDR=0·052) and FEV_1_/FVC (β=–6·0, FDR=0·0084) only in the V–Ex+ event subgroup (appendix p 7).

Cumulative AQI values over 3 days (ie, the day of sample collection and the preceding 2 days) were significantly associated with expression of upper airway gene expression modules that were previously shown to be increased in expression during incipient asthma exacerbations (12 of 19 modules; FDRs <0·05; table 2).^17^ Absolute values of the effect sizes ranged from 0·0015 to 0·0039, where the coefficient represents a change of 0·0015 to 0·0039 in module expression (log2) for a 1 unit increase in AQI over 3 days; expressed differently, this means, for example, a mean 10 unit increase in AQI for 3 days equates to a 3·2–8·5% increase in expression of these modules. A detailed description of these modules was previously published,^17,34^ along with their relationships to V–Ex+ and V+Ex+ events^17^ and details of their contents and annotation.^35^ AQI values were associated with modules that were specifically increased in the V–Ex+ event subgroup but also with core exacerbation modules that were increased in both V+Ex+ and V–Ex+ event subgroups in this population, suggesting a broad effect of air pollution on asthma pathobiological pathways.

Individual pollutant concentrations were also associated with expression of specific modules. PM_2·5_ concentrations were positively associated with modules associated with airway epithelial cell numbers and epithelial gene pathways. These included three modules specific to V–Ex+ events, annotated as tissue kallikrein induction and IL-23–IL-17 axis; keratinisation, epithelial development, cell–cell adhesion, and tight junctions; and squamous epithelium inflammation (FDRs <0·05; appendix pp 8–9). PM_2·5_ concentrations were also pos itively associated with epithelial-cell associated core exacerbation modules upregulated in both V+Ex+ and V–Ex+ events (ie, SMAD3-related cell differentiation, eosinophil activation and mucus hypersecretion, extracellular matrix production and cell membrane, and EGFR signalling and cell–cell adhesion; FDRs<0·05; appendix p 9). The directionality of these associations was not unique to V–Ex+ events but congruent in all four groups. PM_2·5_ concentrations were also associated with the epithelial-cell-associated cilia and IL-33 response module (FDR=0·0087). Absolute values of the effect sizes ranged from 0·006 to 0·014, where the coefficient represents a change of 0·006–0·014 in module expression (log2) for a 1 unit increase in PM_2·5_ over 3 days. For example, the coefficient for the tissue kallikrein induction and IL-23–IL-17 axis module is 0·011, which indicates a 10 μg/m^3^ mean increase in PM_2·5_ for 3 days equates to a 25·7% increase in module expression (appendix p 8). By contrast, O_3_ concentrations were related specifically to increased expression of the type-2 inflammation module (FDR <0·05) with an effect size of 3·76, which indicates a mean 0·05 ppm increase in O_3_ for 3 days equates to a 47·8% increase in module expression (appendix p 8).

We next sought to replicate the finding of increased air pollution concentrations in the V–Ex+ event subgroupnon in an independent cohort. The ICATA study included children, adolescents, and young adults (ie, aged 6–20 years) living in urban areas with persistent allergic asthma (appendix pp 12–13). Nasal samples were collected during 100 asthma exacerbations in the ICATA cohort, 53 of which were virus positive (V+Ex+) and 47 of which were virus negative (V–Ex+). Serving as non-exacerbation comparison timepoints, 165 samples were collected in the absence of respiratory symptoms during a scheduled visit at week 48 of the study, 53 of which were virus positive (V+ at week 48) and 112 of which were virus negative (V– at week 48). Comparing among these four groups, increased AQI, O_3_, and PM_2·5_ values were specifically observed during the V–Ex+ events (p values <0·0001; appendix p 10). NO_2_ was not significant in this analysis (p=0·99 comparing V+Ex+ and V–Ex+). V+Ex+ and V–Ex+ events were not significantly different by city of residence or season (appendix pp 12–13). Increased AQI in the V–Ex+ event subgroup was still significant when adjusted for city of residence (p<0·0001) or for season (p=0·019).

## Discussion

The causes and molecular mechanisms responsible for asthma exacerbations during non-viral illnesses are not fully understood. Here, we identify associations between outdoor air pollution and several distinct airway inflammatory pathways in children living in urban areas with asthma exacerbations during non-viral illnesses. Our findings suggest that air pollutants are associated with asthma exacerbations in dense and impoverished urban communities. The proportion of asthma exacerbations occurring in the absence of a viral illness compared with those occurring with a viral illness is higher than has been observed in paediatric populations with asthma in non-urban areas, in whom only 10–15% of exacerbations occur without detectable virus.^36–38^ However, the high proportion of non-viral events is consistent with past observations of respiratory illnesses in infants from impoverished urban areas (96 [33%] of 295) compared with suburban (63 [11%] of 586).^39^ Furthermore, our findings suggest that moderate increases in local air pollution relative to the US national air quality standards adversely affect these susceptible populations. This association suggests either that exposure to mixtures of pollutants over several days at low concentrations can trigger exacerbations or that high AQI levels exist in these urban communities but are not well captured by regionally reported AQI values on the basis of EPA monitors and public data.

Our study linked individual pollutants to both biological pathways of airway inflammation and pulmonary physiological responses. Specifically, we identified that PM_2·5_ is associated with transcriptional changes of airway epithelial cells for both V+ and V– events, including increases of tissue kallikreins and non-type-2 inflammatory cytokine genes^40^ and a large set of epithelial barrier function genes, which are unique to V–Ex+ events.^17^ The results suggest that increases in PM_2·5_ concentrations trigger many epithelial immune pathways, which might provoke an exacerbation in the absence of a respiratory virus. This inference is consistent with the observed peak of PM_2·5_ in the first several days of V–Ex+ events. Furthermore, PM_2·5_ was associated with pathways that are functionally linked to airway remodelling, including TGFβ signalling, SMAD3 signalling, EGFR signalling, mucus hypersecretion, and extracellular matrix production. These results are consistent with histological and animal model data supporting particulate air pollution as a driver of airway remodelling in part through TGFβ^41,42^ and mucus production.^43,44^ Notably, the directionality of these associations was not unique to V–Ex+ events but congruent in all four groups, suggesting that the airway effects of PM_2·5_ exposure were present in each respiratory event type but that the degree of exposure and hence magnitude of transcriptional change of these modules was highest, and presumably clinically most consequential, in the V–Ex+ group.

We observed that O_3_ is associated with eosinophilic or type-2 inflammation; it was positively associated with expression of a module containing canonical type-2 cytokines *IL4*, *IL5*, and *IL13*, among numerous other elements of type-2 inflammation, and with a concurrent decrease in lung function. This association is consistent with data from studies in animals and humans showing that O_3_ exposure can increase airway eosinophils and induce airway hyper-responsiveness.^11,45^ O_3_ concentrations appeared to be higher for the week leading up to V–Ex+ events, and to a lesser extent V+Ex+ events, than for Ex– events during this period, which could help to explain the observation of sustained type-2 inflammation during V+Ex+ and V–Ex+ events.^17^ Supporting evidence has linked air pollution to asthma symptoms, and the biological plausibility that PM_2·5_ and O_3_ exert their effects through the observed airway inflammatory mech anisms is supported by animal and in-vitro models^46^ and gene-by-environment studies.^47,48^

Our study was observational in nature and had notable limitations. The use of regional pollution data cannot fully characterise individual outdoor pollution exposures compared with, for example, monitoring personal pollution exposure.^49^ There was probably a range in the number and degree of pollutant exposures even among individuals living in regions with identical regional pollutant concentrations, which should be a focus of future studies. We cannot definitively establish that the measured pollutants triggered the airway transcriptome responses in the development of asthma exacerbations given the observational nature of the study. It is possible that unmeasured covariates could also be associated with V–Ex+ events, most notably non-viral infections, indoor pollutants, and inhaled allergens. In a separate analysis of this cohort, we did not find upper airway bacterial or fungal microbes as a cause of the V–Ex+ events, but instead observed that the nasal microbiome showed seasonal dynamics that might predispose to viral infections in the autumn.^50^ We were unable to measure indoor air pollutants during this study, although other studies have shown outdoor air pollution as an important source of indoor air pollution in low-income homes.^51,52^ We noted non-significant increases in V–Ex+ events in the spring and summer months relative to V+Ex+ events, suggesting that seasonal inhaled allergens might also have contributed to V–Ex+ events, but we did not measure inhaled allergen exposures in this study and were able to adjust only for season and site in our analyses, which did not affect our findings. Adjusting for season and site does not entirely address the potential role of inhaled allergen as a contributor to asthma exacerbations in our study. Future studies that can accurately measure acute allergen exposures will be needed to understand their relative contributions. Our study focused on children with persistent asthma with a type-2 phenotype component (ie, peripheral blood eosinophils ≥150/mm^3^ in MUPPITS1 and aeroallergen sensitisation in ICATA). Although we cannot definitively conclude that the results would be generalisable to any asthma phenotype, these results are also highly relevant to type-2 low asthma (ie, often defined as eosinophils <150/mm^3^), in which respiratory irritants might have an even greater role than in persistent asthma with a type-2 high phenotype component.^53^ Our sample size for V– Ex+ events was small in our primary dataset (n=14), and thus showing a similar association of pollution to non-viral illnesses with exacerbations in an independent dataset was an important validation, although the ICATA cohort did not have data to validate the transcriptome results. Finally, this study did not have sufficient power to analyse the potential combined effects of more than one asthma trigger. We hypothesise that viruses, pollution, and other covariates can act independently or synergise to initiate asthma exacerbations in children living in urban areas. In fact, the effects of pollutants might overlap with the effects of viruses, as the associations between several module expression levels and pollutants were congruent in all four event groups. Notably, our data showed combined increases of O_3_ and PM_2·5_ in the V–Ex+ event subgroup, but each pollutant was associated with distinct inflammatory pathways. These pollutants are chemically coupled, can increase concomitantly, especially in warm weather,^54^ and can have additive deleterious effects on the airway. Larger studies will be needed to investigate combined effects among asthma triggers and mechanisms.

Overall, the insights of this study can inform novel asthma management strategies in children living in urban areas. Future strategies could include preventive use of personal air quality monitors and air filters^55^ around periods of risk (ie, when AQI values or concentrations of O_3_ and PM_2·5_ are predicted to be in the moderate or higher EPA categories for a sustained period of a couple of days or more), treatment approaches to counteract deleterious effects of pollutants on the airway epithelium, and targeted therapies aimed at the kallikrein–kinin system or other epithelial pathways and cytokines identified in these modules, in addition to current treatments for type-2 inflammation. Importantly, these data add to the growing body of evidence supporting the need to reduce outdoor air pollution^16,56^ as a means to decrease respiratory illnesses and asthma-related morbidity in children living in urban areas.^57^

## Supplementary Material

MMC1

## Figures and Tables

**Table 1:** MUPPITS1 demographic and clinical characteristics

	Ex+ events			Ex− events		
	V+Ex+ (33 [21·4%] of 154 events; 33 participants)	V-Ex+ (14 [9·1%] of 154 events; 12 participants)	p value	V+Ex− (69 [44·8%] of 154 events; 58 participants)	V-Ex− (38 [24·7%] of 154 events; 34 participants)	p value

Age at baseline, years	12 (10 to 14)	12 (9 to 14)	0·96	9 (8 to 12)	12 (9 to 13)	0·028
Sex	..		0.28	..	..	0·65
Female	15 (45%)	9 (64%)	..	33 (48%)	20 (53%)	..
Male	18 (55%)	5 (36%)	..	36 (52%)	18 (47%)	..
Site	..	..	014*	..	..	0·69*
Boston, MA	1 (3%)	4 (29%)	..	10 (14%)	7 (18%)	..
Chicago, IL	3 (9%)	0	..	4 (6%)	1 (3%)	..
Cincinnati, OH	3 (9%)	0	..	4 (6%)	4 (11%)	..
Dallas, TX	1 (3%)	0	..	6 (9%)	3 (8%)	..
Denver, CO	6 (18%)	5 (36%)	..	8 (12%)	1 (3%)	..
Detroit, MI	6 (18%)	1 (7%)	..	8 (12%)	6 (16%)	..
New York, NY	5 (15%)	3 (21%)	..	18 (26%)	7 (18%)	..
Saint Louis, MO	4 (12%)	0	..	9 (13%)	7 (18%)	..
Washington, DC	4 (12%)	1 (7%)	..	2 (3%)	2 (5%)	..
Season of respiratory symptoms	..	..	0·50	..	..	0·065
Autumn (September-November)	11 (33%)	4 (29%)	..	22 (32%)	3 (8%)	..
Winter (December-February)	12 (36%)	3 (21%)	..	26 (38%)	14 (37%)	..
Spring (March-May)	7 (21%)	2 (14%)	..	17 (25%)	16 (42%)	..
Summer (June-August)	3 (9%)	5 (36%)	..	4 (6%)	5 (13%)	..
Race and ethnicity	..	..	0·75	..	..	0·30
Black	23 (70%)	6 (43%)	..	31 (45%)	22 (58%)	..
Hispanic	8 (24%)	7 (50%)	..	29 (42%)	10 (26%)	..
Other	2 (6%)	1 (7%)	..	9 (13%)	4 (11%)	..
BMI percentile	79 (47 to 95)	84 (46 to 99)	0·32	88 (73 to 97)	85 (64 to 99)	0·96
FEV_1_% predicted	71 (59 to 83)	71 (64 to 80)	0·94	92 (84 to 102)	90 (83 to 104)	0·84
FEV_1_/FVC	0·66 (0·60 to 0·79)	0·6 (0·60 to 0·72)	0·74	0·79 (0·70 to 0·84)	0·78 (0·72 to 0·84)	0·64
FEV_1_ percentage change from baseline	−16 (−27 to 3)	−9 (−29 to −1)	0·71	0 (−9 to 7)	2 (−11 to 9)	0·92
FEV_1_/FVC change from baseline	−0·07 (−0·13 to 0·00)	−0·06 (−0·14 to 0·00)	0·99	0·01 (−0·04 to 0·05)	0·01 (−0·04 to 0·03)	0·52
Fractional exhaled NO	39 (21 to 58)	30 (14 to 100)	0·66	35 (15 to 65)	30 (16 to l2)	0·61
Urine cotinine concentrations†	..	..	0·61	..	..	0·35*
Level 0 (0–10 ng/mL)	7 (21%)	4/13 (31%)	..	12 (17%)	6/35 (17%)	..
Level 1 (10–30 ng/mL)	14 (42%)	3/13 (23%)	..	39 (57%)	18/35 (51%)	..
Level 2 (30–100 ng/mL)	10 (30%)	4/13 (31%)	..	8 (12%)	8/35 (23%)	..
Level 3 (100–200 ng/mL)	2 (6%)	2/13 (15%)	..	9 (13%)	2/35 (6%)	..
Level 4 (200–500 ng/mL)	0	0	..	0	1/35 (3%)	..
Level 5 (500–1000 ng/mL)	0	0	..	1 (1%)	0/35	..
Respiratory illness severity by WURSS AUC over 10 days starting at respiratory illness onset	44 (36 to 61)	38 (22 to 82)	0·47	38 (19 to 59)	28 (17 to 42)	0·070
Time between respiratory illness onset and corticosteroid use, days	2 (2 to 3)	4 (2 to 6)	0·27	NA	NA	NA
Duration of exacerbation, days	4·0 (4·0 to 8·0)	4·0 (4·0 to 4·8)	0·44	NA	NA	NA
Reported time with missed inhaled corticosteroid dose in the 14 days preceding the reported respiratory illness onset, days	0·0 (0·0 to 2·0)	0·0 (0·0 to 1·0)	0·97	0·0(0·0 to 2·0)	0·0 (0·0 to 2·0)	0·65
Number of inhaled corticosteroid doses counted in the past 14 days	28 (23 to 28)	26 (24 to 28)	0·47	26 (22 to 28)	26 (23 to 28)	0·81
Number of positive serum-specific IgE	7 (4 to 10)	6 (4 to 12)	0·72	5 (3 to 10)	10 (5 to 11)	0·040
Number of positive aeroallergen skin tests	6 (3 to 6)	7 (4 to 10)	0·067	4 (2 to 6)	4 (2 to 7)	0·89
Sensitised to at least one aeroallergen	33 (100%)	14 (100%)	1·0	69 (100%)	38 (100%)	1·0
Viral respiratory illness type						
Rhinovirus A	11 (33%)	NA	..	14 (20%)	NA	..
Rhinovirus C	9 (27%)	NA	..	21 (30%)	NA	..
Rhinovirus B	1 (3%)	NA	..	8 (12%)	NA	..
Enterovirus	1 (3%)	NA	..	0	NA	..
Rhinovirus or enterovirus (nonspecific)	0	NA	..	2 (3%)	NA	..
Adenovirus	0	NA	..	2 (3%)	NA	..
Bocavirus	1 (3%)	NA	..	7 (10%)	NA	..
Respiratory syncytial virus A or B	2 (6%)	NA	..	3 (4%)	NA	..
Coronavirus‡	4 (12%)	NA	..	11 (16%)	NA	..
Parainfluenza virus	4 (12%)	NA	..	4 (6%)	NA	..
Metapneumovirus	2 (6%)	NA	..	3 (4%)	NA	..
Influenza B	0	NA	..	2 (3%)	NA	..
More than one virus	2 (6%)	NA	..	8 (12%)	NA	..

Data are median (IQR) or n (%) and represent the number of events. Events are the number of unique illness events. Participants are the number of unique participants with illness events. For participants with two respiratory illnesses meeting specified criteria, both illnesses are included in the table. Summaries apply to the first visit during that illness. p values represent the significance of any difference between the proportion of V+Ex+ versus V–Ex+ or V+Ex– versus V–Ex–. All p values are from generalised linear mixed-effect models with a random effect for participant to account for correlation between values from the same participant, except for instances where at least one of the categories of a categorical variable is zero and p values are from a Fisher’s exact test. Ex+=exacerbation. Ex–=non-exacerbation. V+Ex+=viral event with exacerbation. V–Ex+=non-viral event with exacerbation. V+Ex–=viral event without exacerbation. V–Ex–=non-viral event without exacerbation. FEV_1_% predicted=FEV_1_ as a percentage of predicted. FEV_1_/FVC=ratio of FEV_1_ to forced vital capacity. FEV_1_=forced expiratory volume in 1s. WURSS=Wisconsin Upper Respiratory Symptom Survey for kids—daily symptom report. AUC=area under the curve. NA=not applicable.

* p values are from a Fisher’s exact test.

† Urine was not collected for one V–Ex+ event and three V–Ex–events.

‡ Seasonal, non-SARS coronaviruses.

**Table 2:** Gene expression modules differentially expressed during asthma exacerbations

	Cell association	Number of genes	AQI FDR	AQI effect size	PM_2.5_ FDR	PM_2.5_ effect size	O_3_ FDR	O_3_ effect size

**V-Ex+ specific modules**								
Keratinisation, epithelial development, cell–cell adhesion, and tight junctions	Epithelium	1041	0·023	0·0032	0·0060	0·0137	0·44	2·0302
Tissue kallikreins and IL-23–IL-17 axis	None	143	0·074	0·0023	0·019	0·0108	0·83	0·4875
Squamous epithelium	Epithelium	56	0·0092	0·0019	0·018	0·0057	0·44	1·0821
**Core modules**								
SMAD3-related cell differentiation	Epithelium	46	<0·0001	0·0038	0·0008	0·0115	0·42	1·7530
Eosinophil activation and mucus hypersecretion	Eosinophil and epithelium	69	0·0092	0·0037	0·012	0·0116	0·10	4·0189
Extracellular matrix production and cell membrane	Epithelium	209	0·0092	0·0034	0·0035	0·0129	0·42	2·4324
EGFR signalling and cell–cell adhesion	Epithelium	240	0·0092	0·0035	0·0035	0·0134	0·42	2·8261
Lymphocyte proliferation	Lymphocyte	266	0·49	–0·0008	0·72	–0·0017	0·59	–1·1506
B-cell receptor signalling	Lymphocyte	378	0·91	–0·0001	0·97	0·0001	0·83	–0·3962
**V-Ex+ specific modules**								
Type-1 interferon response	None	262	0·012	0·0036	0·054	0·0098	0·56	1·7208
Chemoattraction and cytotoxicity	Macrophage	247	0·011	0·0039	0·062	0·0098	0·44	2·1856
Heat shock proteins or stress response	None	65	0·054	0·0020	0·59	0·0023	0·59	1·0395
Cilia and IL-33 response	None	409	0·074	0·0026	0·0087	0·0143	0·65	1·2294
Antigen-presenting cell co-stimulation	Lymphocyte and macrophage	58	0·080	0·0017	0·20	0·0045	0·59	0·9560
Antigen processing and presentation	Macrophage	283	0·011	0·0019	0·12	0·0039	0·42	1·3168
Unannotated*	Macrophage	65	0·13	0·0013	0·22	0·0038	0·44	1·3166
Type-2 inflammation	Eosinophil	242	0·039	0·0020	0·73	0·0012	0·037	3·7624
Chromatin modification and regulation of gene expression	None	144	0·039	–0·0016	0·15	–0·0040	0·83	–0·3036
Protein catabolism	None	495	0·023	–0·0015	0·10	–0·0038	0·91	–0·0946

Expression of some modules was significantly associated with AQI values, PM_2·5_ concentrations, and O_3_ concentrations. Module expression was compared with AQI and pollutant values summed over the two preceding days and day of nasal sample collections (V–Ex+ n=17, V+Ex+ n=38, V–Ex– n=72, V+Ex– n=120). Significance was determined by generalised linear mixed effect models with a random effect for participant. Results were considered significant if the FDR was <0·05. AQI=air quality index. FDR=false discovery rate. NS=not significant. NA=not applicable. V–Ex+=non-viral event with exacerbation. V+Ex+=viral event with exacerbation.

* Biology could not be interpreted.

## Research in context

### Evidence before this study

Respiratory viral infections are the most common cause of asthma exacerbations in children and adolescents (ie, aged <21 years), and human studies of respiratory viruses in asthma have identified key inflammatory pathways mediating their effects. These findings have increasingly led towards targeted therapeutic strategies to prevent asthma exacerbations. Asthma exacerbations also occur independently of viral respiratory infections, but the causes and molecular mechanisms of non-viral exacerbations are, in comparison, poorly understood. Over the past several decades, many epidemiological studies have shown associations between air pollutant concentrations and the occurrence of asthma exacerbations. Mechanistic animal, cellular, and controlled human studies have suggested plausible mechanisms by which individual air pollutants can cause airway inflammation. However, studies linking air pollutant exposures to molecular mechanisms in the airways during naturally occurring asthma exacerbations have not been conducted. We searched PubMed for articles published in English between Jan 1, 2010, and Oct 1, 2021, using the terms “asthma exacerbation” AND “air pollution”, restricted to human studies. We also reviewed all references from two in-depth review articles on the topic of environmental exposures, outdoor air pollution, and asthma, published in 2014 and 2019.

### Added value of this study

We identify that air quality in urban centres, related to increased concentrations of specific air pollutants, is significantly associated with asthma exacerbations in children who are underprivileged and living in areas with high disease burden. We show that air pollutants are associated with exacerbations occurring in the absence of a provoking respiratory virus. Crucially, we connect individual pollutants to changes in airway physiology and upper airway gene expression during acute exacerbations, identifying associations with decreased lung function and distinct molecular pathways of airway inflammation. We link fine particulate matter (PM_2·5_) exposure to several epithelial inflammatory responses, including induction of tissue kallikreins and inflammatory cytokines, which are absent in viral-induced exacerbations, and to airway remodelling pathways. Similarly, we link O_3_ exposure to type-2 inflammatory responses and decreased lung function. For the first time, to our knowledge, we identify associations between specific pollutant exposures and airway inflammatory patterns that are present in non-viral asthma exacerbations, contributing to asthma disparities in children living in urban settings.

### Implications of all the available evidence

Regional air pollutants are epidemiologically associated with asthma exacerbations and might represent an important trigger of asthma exacerbations occurring independently of respiratory viral infections. Compared with viral exacerbations, non-viral exacerbations associated with air pollution proceed through distinct and overlapping molecular mechanisms and also share some common mechanisms. This increased understanding of the potential causes and distinct molecular mechanisms of asthma exacerbation endotypes can inform targeted interventional strategies and bolster the public health argument for policies that reduce outdoor air pollution.
