# Supplementary material for: Associations between outdoor air pollutants and non-viral asthma exacerbations and airway inflammatory responses in children and adolescents living in urban areas in the USA: a retrospective secondary analysis
Source: Lancet Planet Health. Author manuscript; Available in PMC 2023 Mar 3. (PMC9984226; doi:10.1016/S2542-5196(22)00302-3)
Supplement: MMC1 [file NIHMS1863610-supplement-MMC1.pdf]

THE LANCET
Planetary Health

Supplementary appendix

This appendix formed part of the original submission and has been peer reviewed. We post it as supplied by the authors.

Supplement to: Altman MC, Kattan M, O'Connor GT, et al. Associations between outdoor air pollutants and non-viral asthma exacerbations and airway inflammatory responses in children and adolescents living in urban areas in the USA: a retrospective secondary analysis. *Lancet Planet Health* 2023; **7:** e33–44.

Supplementary Materials

Relationships of outdoor air pollutants to non-viral asthma exacerbations and airway inflammatory responses in urban children and adolescents: a population-based study

Table of Contents:

Title Page	1
Supplementary Figures	2-10
Supplementary Tables	11-13

Authors:
Matthew C Altman MD, Prof Meyer Kattan MD, Prof George T O'Connor MD, Ryan C Murphy MD, Elizabeth Whalen PhD, Petra LeBeau PhD, Agustin Calatroni MS, Prof Michelle A Gill MD PhD, Prof Rebecca S Gruchalla MD PhD, Prof Andrew H Liu MD, Stephanie Lovinsky-Desir MD, Prof Jacqueline A Pongracic MD, Carolyn M Kercsmar MD, Prof Gurjit K Khurana Hershey MD PhD, Prof Edward M Zoratti MD, Prof Stephen J Teach MD, Prof Leonard B Bacharier MD, Lisa M Wheatley MD, Steve M Sigelman RN, Peter J Gergen MD, Alkis Togias MD, Prof William W Busse MD, Prof James E Gern MD, Prof Daniel J Jackson MD on behalf of the National Institute of Allergy & Infectious Disease's Inner City Asthma Consortium

Figure S1 **AQI values are higher in relation to Ex+ illnesses than Ex- illnesses.**

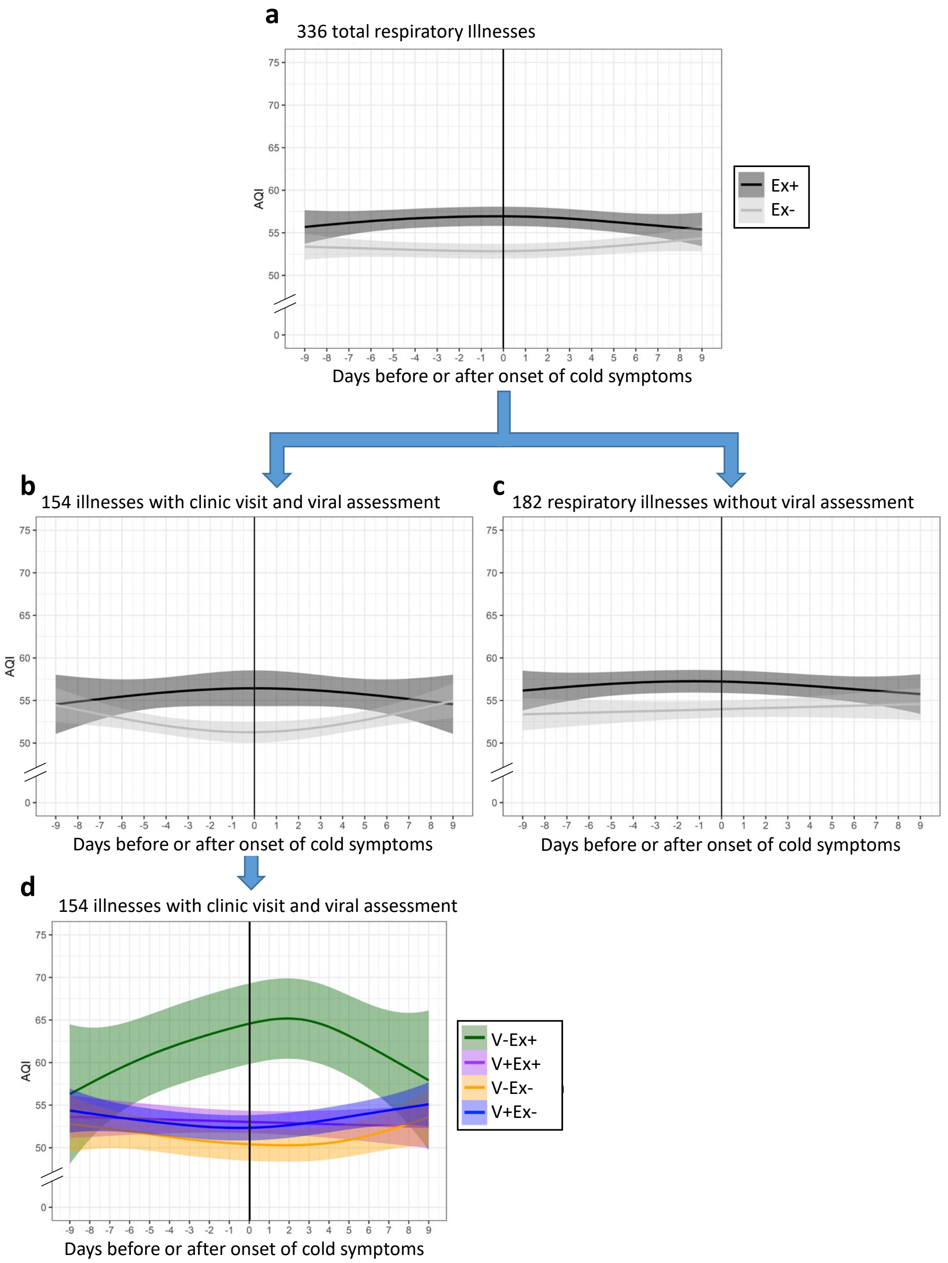

a, The longitudinal levels of AQI in Ex+ and Ex- illness subgroups for the 336 illnesses show AQI levels were elevated in Ex+ illnesses relative to Ex- illnesses across the timespan of 9 days before the reported start of respiratory symptoms (day 0) to 9 days after ($p<1e-4$). Number of illness events per group: Ex+ n = 143, Ex- n = 193. **b**, Similar results exist in the 154 illnesses with detailed assessment including virology data ($p<1e-4$) (number of illness events per group: Ex+ n = 47, Ex- n = 107) and **c**, in the 182 other illnesses lacking virology data ($p<1e-4$) (number of illness events per group: Ex+ n = 96, Ex- n = 86). **d**, The elevated AQI signal is seen to be pronounced in the V-Ex+ illnesses ($p<1e-4$) (same as Figure 1a) (number of illness events per group: V-Ex+ n = 14, V+Ex+ n = 33, V-Ex- n = 38, V+Ex- n = 69). Longitudinal data were plotted using GAM fits showing 95% confidence intervals.

<u>Figure S2</u> **AQI values and individual pollutant levels were elevated before and during non-viral asthma exacerbations.**

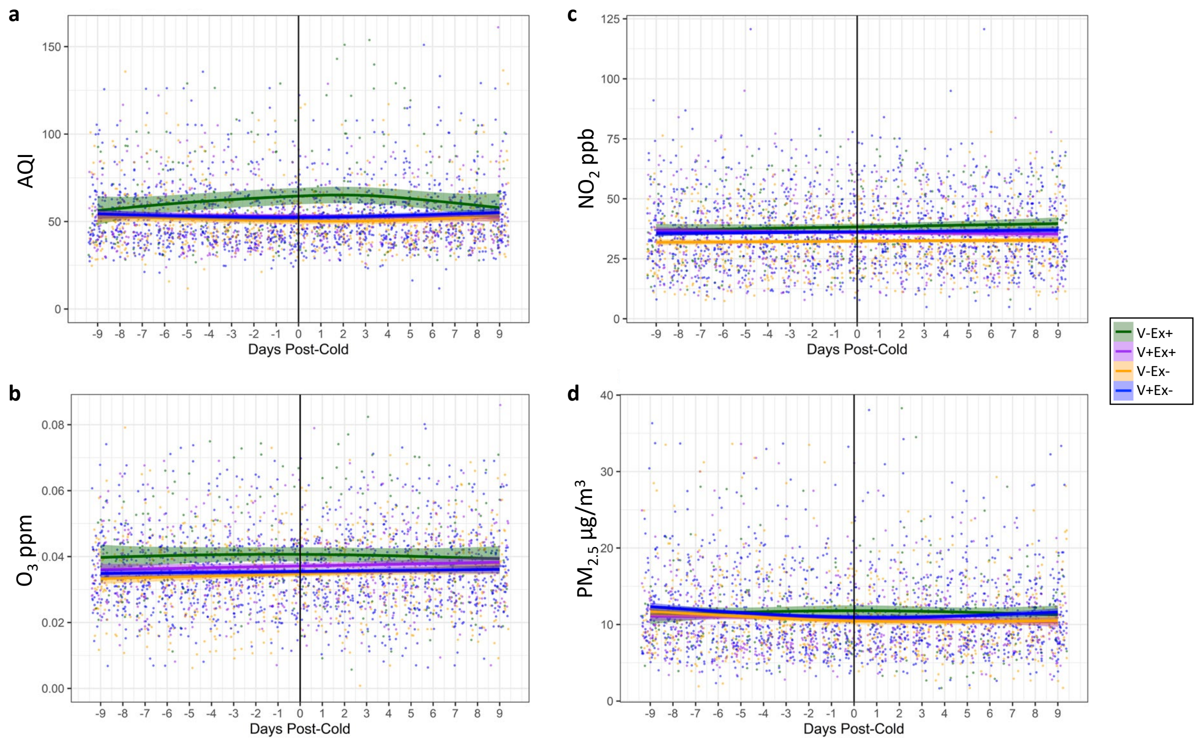

These plots are equivalent to Figure 1a,c,d,e except that they display all data points and have expanded y-axes to show the full spread of the data. **a**, The longitudinal levels of AQI in each of the four illness event subgroups show AQI values were elevated during the V-Ex+ illnesses relative to the other three illness subgroups across the timespan of 9 days before the reported start of respiratory symptoms (day 0) to 9 days after ($p<1e-4$). **b**, The longitudinal levels of O_3 were elevated in the V-Ex+ illnesses relative to the other three illness groups ($p<1e-4$) and also modestly elevated in the V+Ex+ illness group relative to the Ex- groups ($p=1.4E-3$). **c,d**, The longitudinal levels of NO_2 ($p<1e-4$) and $PM_{2.5}$ ($p=6.0E-4$) were elevated in the V-Ex+ illnesses relative to the other three illness groups. Longitudinal data were plotted using GAM fits showing 95% confidence intervals; all data points are shown. Number of illness events per group: V-Ex+ n = 14, V+Ex+ n = 33, V-Ex- n = 38, V+Ex- n = 69.

<u>Figure S3</u> **Case crossover comparisons show AQI values are elevated in the week leading up to a non-viral exacerbation relative to the prior week, with similar trends for individual pollutant levels.**

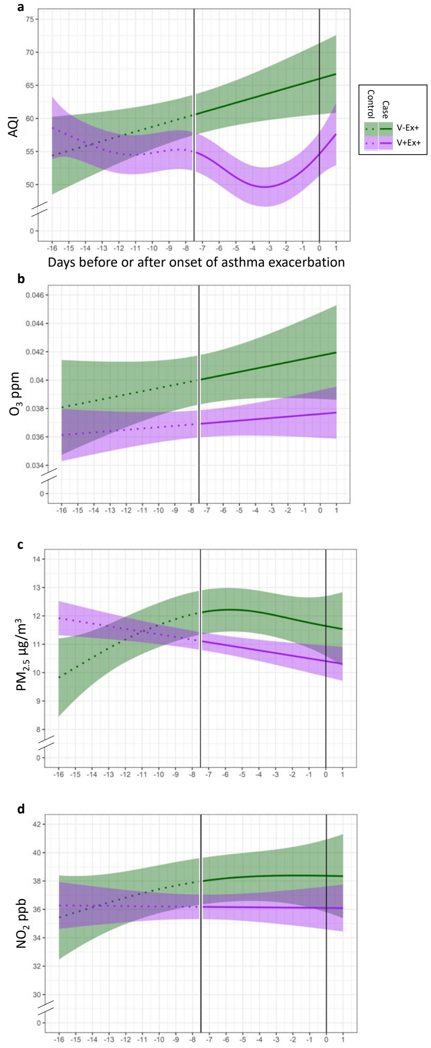

a, Shown are the longitudinal levels of AQI in V-Ex+ and V+Ex+ illness subgroups now timed in relation to the start of an asthma exacerbation (day 0, vertical black line). The results show AQI levels are increased in the ~1 week leading up to a V-Ex+ illness (days -7 to +1; solid green line right of vertical gray line) compared to the prior period (days -16 to -8; dotted green line left of vertical gray line) (p=7.5e-3). This is not the case in V+Ex+ (purple). Similar to Figure 1a, this analysis confirms the higher levels of AQI in V-Ex+ compared to V+Ex+ illnesses during this period (days -16 to +1; p<1e-4). That difference is specifically driven by days -7 to +1 (p<1e-4) rather than days -16 to -8 (p=0.68). **b**, A trend can be seen for O_3: V-Ex+ illness days -7 to +1 are non-significantly higher compared to the days -16 to -8 (green) (p=0.16). Additionally this analysis confirms the higher O_3 levels in V-Ex+ compared to V+Ex+ illnesses during this period days -16 to +1 (p=1.1e-3), driven by days -7 to +1 (p=4.3e-3) more so than days -16 to -8 (p=7.5e-2). **c**, A trend can be seen for $PM_{2.5}$: V-Ex+ illness days -7 to +1 are borderline significantly higher compared to the days -16 to -8 (green) (p=0.10). Additionally this analysis confirms the higher $PM_{2.5}$ levels in V-Ex+ compared to V+Ex+ illnesses during this period specifically driven by days -7 to +1 (p=2.4e-2) rather than days -16 to -8 (p=0.33). **d**, A trend can be seen for NO_2: V-Ex+ illness days -7 to +1 are non-significantly higher compared to the days -16 to -8 (green) (p=0.17). NO_2 levels in V-Ex+ compared to V+Ex+ illnesses trend towards higher during days -7 to +1 (p=0.087) but not days -16 to -8 (p=0.96). Longitudinal data were plotted using GAM fits showing 95% confidence intervals. Number of illness events per group: V-Ex+ n = 14, V+Ex+ n = 33.

Figure S4 **AQI values were elevated before and during non-viral asthma exacerbation subset to 5 cities.**

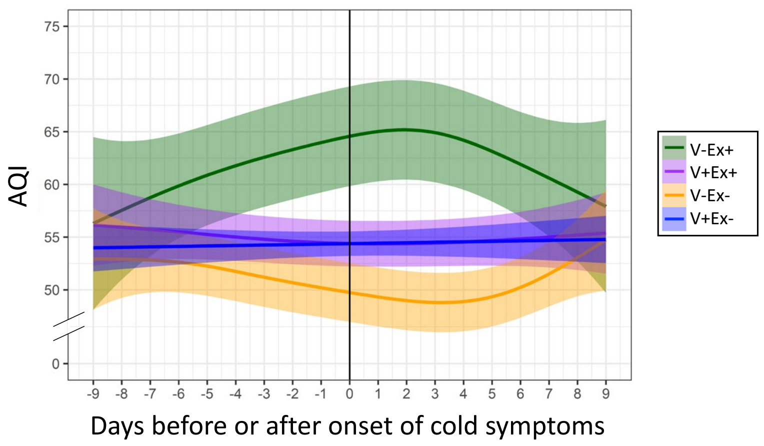

AQI values were elevated before and during non-viral asthma exacerbations after subsetting to illnesses only in the 5 clinical sites where V-Ex+ illnesses were recorded: Boston, New York, Detroit, Denver, and Washington D.C. The longitudinal levels of AQI in each of the four illness event subgroups show AQI levels elevated during the V-Ex+ illnesses relative to the other three illness subgroups across the timespan of 9 days before the reported start of respiratory symptoms (day 0) to 9 days after ($p<1e-4$). Longitudinal data were plotted using GAM fits showing 95% confidence intervals. Number of illness events per group: V-Ex+ n = 14, V+Ex+ n = 22, V-Ex- n = 23, V+Ex- n = 46.

<u>Figure S5</u> **AQI values were elevated before and during non-viral asthma exacerbation subset by indicated seasons.**

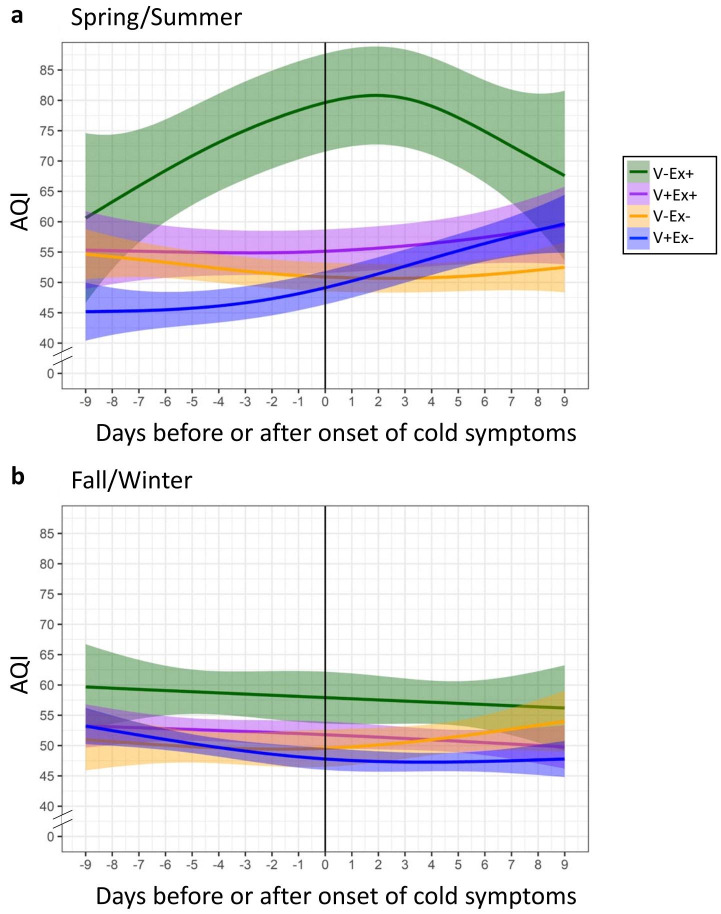

AQI levels were elevated before and during non-viral asthma exacerbations after subsetting to illnesses into **a**, Spring/Summer (p<1e-4) or **b**, Fall/Winter (p<1e-4). The longitudinal levels of AQI in each of the four illness event subgroups show AQI levels elevated during the V-Ex+ relative to the other three illness subgroups across the timespan of 9 days before the reported start of respiratory symptoms (day 0) to 9 days after. Longitudinal data were plotted using GAM fits showing 95% confidence intervals. Number of illness events per group in **a**: V-Ex+ n = 7, V+Ex+ n = 10, V-Ex- n = 21, V+Ex- n = 21. Number of illness events per group in **b**: V-Ex+ n = 7, V+Ex+ n = 23, V-Ex- n = 17, V+Ex- n = 48.

<u>Figure S6</u> **Pulmonary functions were inversely associated with O_3 levels in V-Ex+ illnesses.**

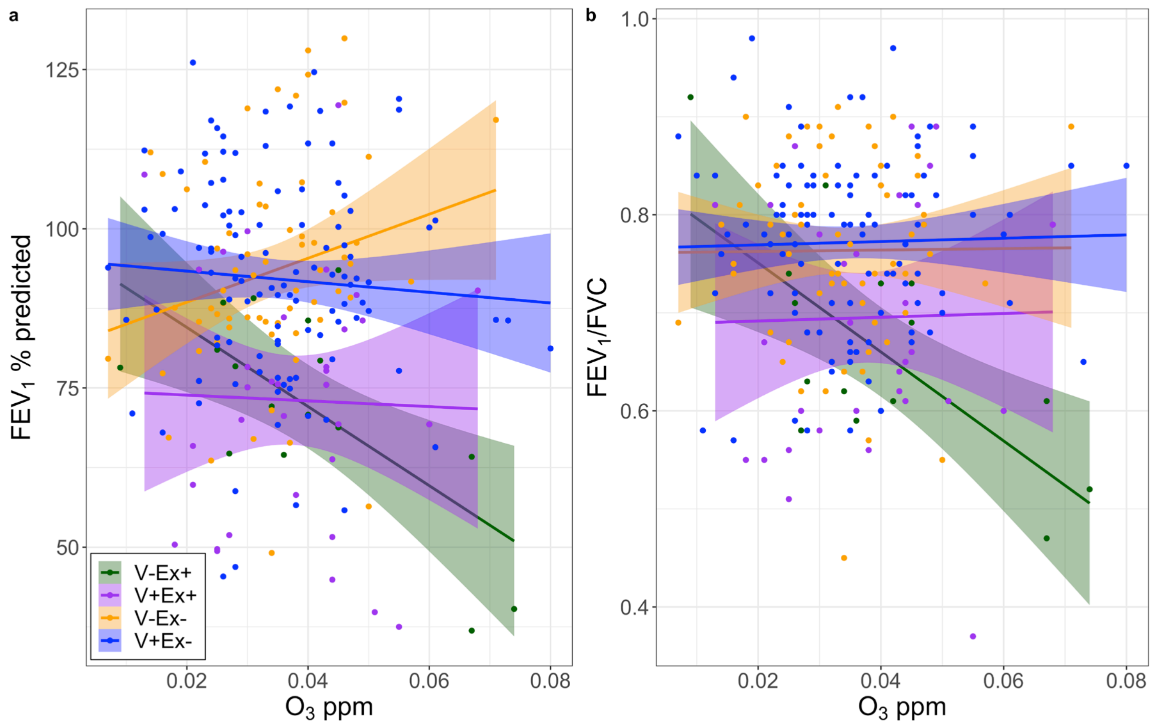

a, The FEV_1 % predicted was inversely associated with the O_3 level measured on the same day in the V-Ex+ illnesses (linear mixed effects model parameter ρ=-591, FDR=0.052) and was not significantly associated in the other 3 illness groups. **b**, The FEV_1/FVC ratio predicted was inversely associated with the O_3 level measured on the same day only in the V-Ex+ illnesses (linear mixed effects model parameter ρ=-6.0, FDR=0.0084) and was not significantly associated in the other 3 illness groups. Shown are regression lines and 95% confidence intervals for each group along with all data points for each group. Number of data points per group: V-Ex+ n = 16, V+Ex+ n = 36, V-Ex- n = 70, V+Ex- n = 116.

Figure S7 **Distinct airway gene expression modules were associated with individual pollutants.**

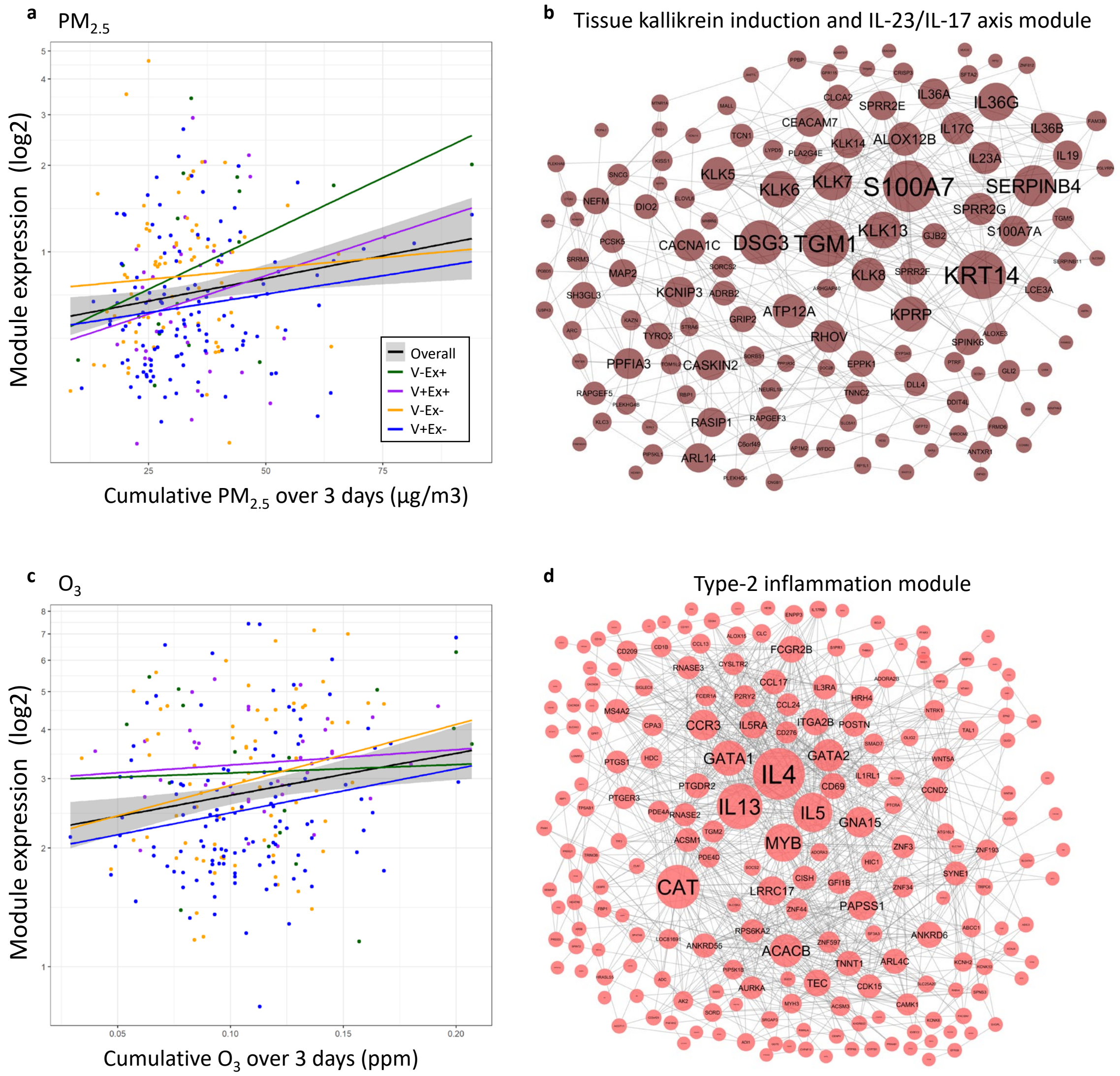

a, The upper airway expression levels of the "Tissue kallikrein induction and IL-23/IL-17 axis" module was significantly associated with $PM_{2.5}$ levels (cumulative over the two preceding days and day of nasal sample collection). **b**, This gene coexpression module is significantly enriched for tissue kallikreins as well as IL-23/IL-17 axis genes. **c**, The upper airway expression levels of the type-2 inflammation module was significantly associated with O_3 levels (cumulative over the two preceding days and day of nasal sample collection). **d**, This gene coexpression module is enriched for type-2 inflammatory molecules. Shown are regression lines for each group, and an overall regression line and 95% confidence interval along with all data points for each group. Number of data points per group: V-Ex+ n = 17, V+Ex+ n = 38, V-Ex- n = 72, V+Ex- n = 120. Gene-gene associations demonstrate significant interaction networks centered around key genes. Gene names are provided accordingly to their HUGO gene nomenclature committee (HGNC) gene symbol and represented in circular nodes, and known gene-gene interactions from STRING are drawn as connecting edges. The size of each node is proportional to the number of interactions. The networks are drawn as force-directed graphs, meaning genes towards the center have the greatest centrality within the network. The summary annotation of each module was previously derived from manual inspection of the module cell correlation, functional enrichment, and interaction network.

Figure S8 **Distinct airway gene expression modules were associated with $PM_{2.5}$ levels.**

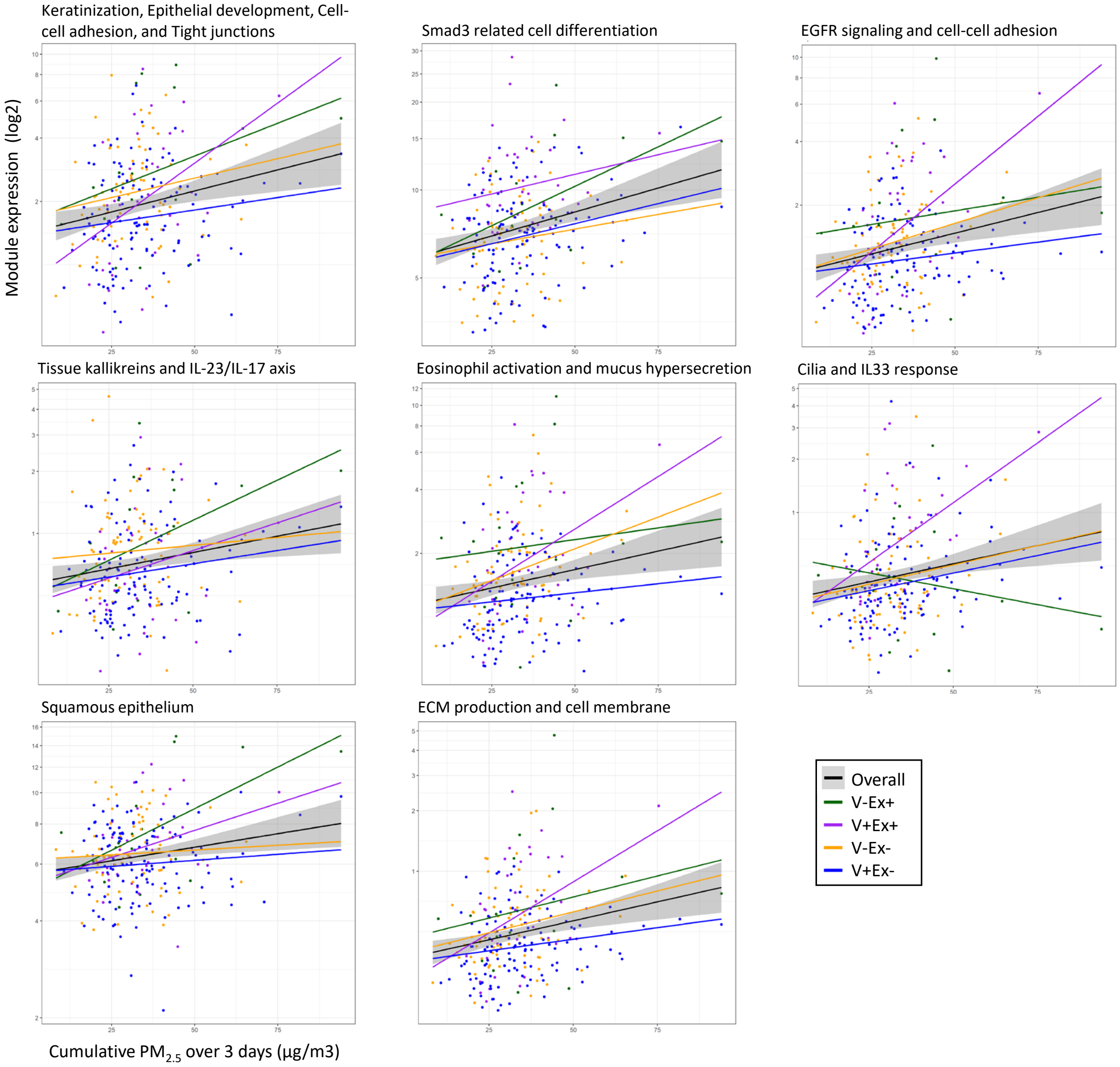

Shown are all significant associations between individual gene expression modules and $PM_{2.5}$ levels (cumulative over the two preceding days and day of nasal sample collection), which are also listed in Table 2. Shown are regression lines for each group, and an overall regression line and 95% confidence interval along with all data points for each group. Number of data points per group: V-Ex+ n = 17, V+Ex+ n = 38, V-Ex- n = 72, V+Ex- n = 120.

Figure S9 **AQI values and individual pollutant levels were elevated before and during non-viral asthma exacerbations.**

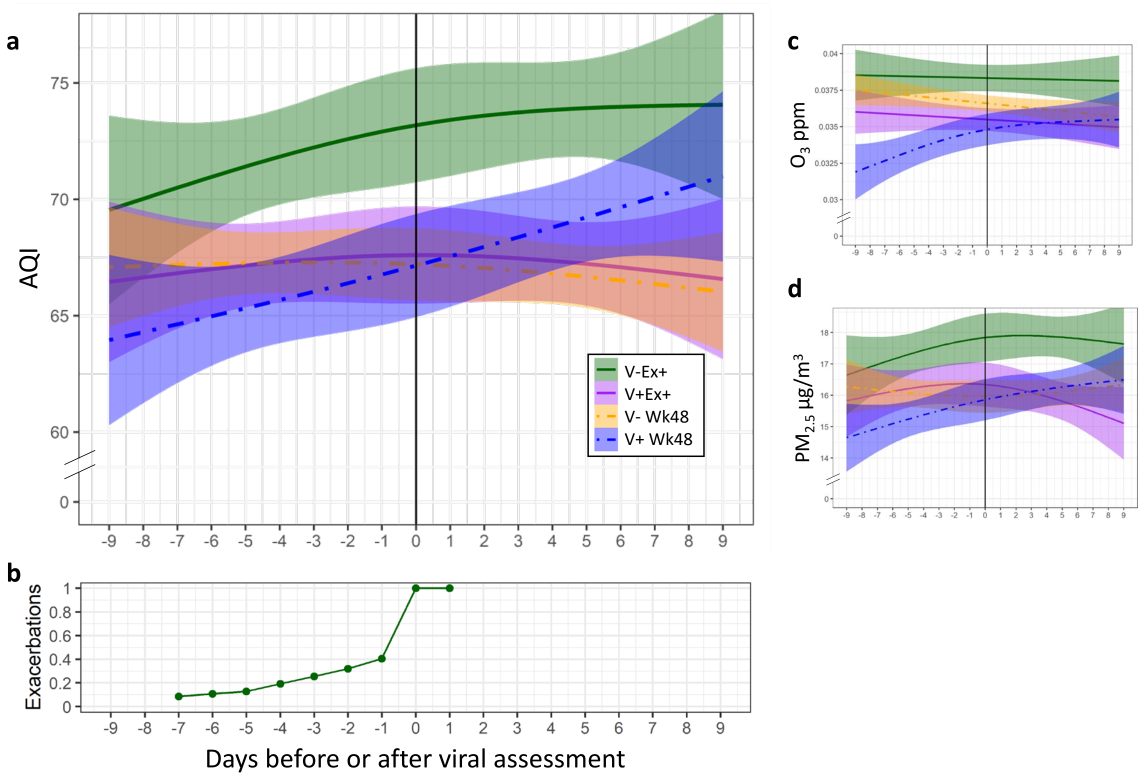

a, The longitudinal levels of AQI in each of the four subgroups from the ICATA study showing AQI values were elevated during the non-viral asthma exacerbation illnesses (V-Ex+, green) relative to the other three groups across the timespan of 9 days before the reported start of respiratory symptoms (day 0) to 9 days after ($p<1E-4$). Day of sample assessment is noted as day 0. **b**, The cumulative incidence of exacerbations in the V-Ex+ subgroup relative to the time of sample collection (day 0). **c,d**, The longitudinal levels of O_3 and $PM_{2.5}$ were elevated in the V-Ex+ illnesses relative to the other three groups ($p<1e-4$). Longitudinal data were plotted using GAM fits showing 95% confidence intervals. Number of illness events per group: V-Ex+ n = 47, V+Ex+ n = 53, V- wk48 n = 112, V+ wk48 n = 53.

Table S1 **MUPPITS1 additional clinical characteristics**

**Characteristic**	Exacerbation Events			Non-exacerbation Events		
	**Ex+Virus+**, N = 33[1]	**Ex+Virus-**, N = 14[1]	**p-value**[2*]	**Ex-Virus+**, N = 69[1]	**Ex-Virus-**, N = 38[1]	**p-value**[2*]
**Nasal Cell Differentials**						
Neutrophils	78.4 (62,90.2)	49.8 (42.2,71)	0.20	97 (91.5,99)	84 (58.5,95.5)	<0.001
Lymphocytes	1.2 (0.6,3.2)	1.4 (0.6,2.6)	0.14	87 (57.4,92.8)	59 (29,83.6)	0.002
Macrophages	4.4 (2.2,10.4)	3.2 (2.6,4.2)	0.15	1.2 (0.6,3)	1.4 (0.2,4)	0.86
Eosinophils	3.2 (1,6.8)	3 (2.2,32.4)	0.15	4.2 (2.6,6.8)	3.6 (1.6,6.6)	0.19
Epithelial cells	3 (1,6)	7 (4,43)	0.40	1.6 (0.2,8.2)	5.8 (0.6,22)	0.05
**Blood Cell Differentials**						
Neutrophils	49.5 (40.8,61)	53.2 (42.6,61)	0.77	54.1 (44,60)	47 (36.8,60)	0.91
Lymphocytes	31.6 (21,38)	30 (27.4,38.2)	0.65	30.5 (25.6,38)	39.3 (28,43)	0.21
Monocytes	8.6 (6.2,12)	6.4 (5.8,7)	0.61	7.7 (6.8,10)	7 (6,8)	0.08
Eosinophils	6.8 (5,9)	7 (4.8,11)	0.04	5.3 (3.2,9.2)	5.5 (3.4,10.2)	0.04
Basophils	0.7 (0.4,1)	0.6 (0,0.8)	0.93	0.6 (0.4,1)	0.8 (0.2,1)	0.41
**# Positive Serum Specific IgE**	7 (4, 10)	6 (4, 12)	0.72	5 (3, 10)	10 (5, 11)	0.04
Sensitive to Alternaria (IgE)	17 (53%)	6 (43%)	0.53	22 (33%)	15 (41%)	0.41
Sensitive to American Cockroach (IgE)	9 (29%)	5 (36%)	0.67	14 (21%)	14 (38%)	0.083
Sensitive to Aspergillus Fumigatus (IgE)	16 (50%)	6 (43%)	0.65	23 (34%)	16 (43%)	0.37
Sensitive to Cat (IgE)	22 (69%)	13 (93%)	0.12	44 (66%)	22 (59%)	0.53
Sensitive to Cladosporium (IgE)	16 (50%)	4 (29%)	0.24	25 (37%)	18 (49%)	0.26
Sensitive to Der f (IgE)	17 (53%)	9 (64%)	0.51	28 (42%)	23 (62%)	0.06
Sensitive to Der p (IgE)	17 (53%)	10 (71%)	0.28	29 (43%)	18 (49%)	0.59
Sensitive to Dog (IgE)	27 (84%)	13 (93%)	0.44	51 (76%)	30 (81%)	0.59
Sensitive to Maple (IgE)	18 (56%)	6 (43%)	0.43	23 (34%)	29 (78%)	<0.001
Sensitive to Mouse (IgE)	12 (38%)	9 (64%)	0.12	30 (45%)	17 (46%)	0.97
Sensitive to Oak (IgE)	15 (47%)	6 (43%)	0.81	29 (43%)	23 (62%)	0.083
Sensitive to Pecan (IgE)	15 (45%)	6 (43%)	0.87	21 (31%)	24 (65%)	0.006
Sensitive to Ragweed (IgE)	14 (44%)	7 (50%)	0.75	21 (31%)	21 (57%)	<0.001
Sensitive to German Cockroach (IgE)	13 (42%)	6 (43%)	0.95	29 (43%)	16 (43%)	0.98
Sensitive to Timothy Grass (IgE)	12 (36%)	6 (43%)	0.69	22 (33%)	18 (49%)	0.13
**# Positive Aeroallergen Skin Tests**	6 (3, 6)	7 (4, 10)	0.067	4 (2, 6)	4 (2, 7)	0.89
Sensitive to Alternaria	14 (45%)	4 (33%)	0.49	18 (28%)	8 (22%)	0.58
Sensitive to American Cockroach	11 (35%)	8 (67%)	0.072	26 (40%)	14 (39%)	0.93
Sensitive to Aspergillus Fumigatus	3 (10%)	3 (25%)	0.22	11 (17%)	4 (11%)	0.43
Sensitive to Bermuda Grass	4 (36%)	4 (67%)	0.24	8 (50%)	2 (33%)	0.51
Sensitive to Cat	17 (57%)	8 (67%)	0.56	31 (47%)	12 (32%)	0.14
Sensitive to Der f	17 (53%)	8 (67%)	0.43	24 (36%)	13 (36%)	<0.001
Sensitive to Der p	12 (39%)	6 (50%)	0.51	23 (35%)	13 (35%)	0.98
Sensitive to Dog	14 (45%)	6 (50%)	0.79	14 (21%)	4 (11%)	0.19
Sensitive to KOT Grass Mix	11 (34%)	5 (42%)	0.67	18 (27%)	14 (38%)	0.26
Sensitive to Juniper	4 (44%)	4 (80%)	0.27	3 (17%)	2 (25%)	0.63
Sensitive to Mouse	14 (44%)	7 (58%)	0.40	26 (38%)	16 (42%)	0.73
Sensitive to Ragweed	9 (30%)	4 (33%)	0.84	17 (27%)	14 (39%)	0.23
Sensitive to Rat	11 (35%)	6 (50%)	0.39	23 (35%)	11 (30%)	0.57
Sensitive to German Cockroach	13 (42%)	9 (75%)	0.061	25 (38%)	19 (51%)	0.21
Sensitive to East 8 Tree Mix	17 (53%)	8 (67%)	0.43	29 (43%)	18 (49%)	0.59

[1] Statistics presented: Median (IQR); n (%)
Note: For subjects with 2 colds meeting specified criteria, both colds are included in the table. Summaries apply to the first visit during that illness.
[2,] Statistical tests performed: All p-values are from generalized linear mixed effect models with a random effect for participant ID to account for correlation between values from the same participant, except for instances where at least one of the categories of a categorical variable is zero and p-values are from a Fisher's Exact Test (indicated with [*]).*

Table S2 **ICATA demographic and clinical characteristics**

**Characteristic**	Exacerbation Events			Non-exacerbation scheduled samples		
	**Ex+Virus+**, N = 53[1]	**Ex+Virus-**, N = 47[1]	**p-value**[2]	**Ex-Virus+**, N = 53[1]	**Ex-Virus-**, N = 112[1]	**p-value**[2]
Age (years) at Baseline	9 (7, 12)	11 (8, 14)	0.10	9 (7, 12)	11 (8, 14)	0.02
Gender - Female	17 (32%)	18 (38%)	0.53	19 (36%)	44 (39%)	0.67
Site			0.56			0.72
Chicago	11 (21%)	8 (17%)		12 (23%)	26 (23%)	
Cleveland	7 (13%)	8 (17%)		8 (15%)	25 (22%)	
Dallas	13 (25%)	7 (15%)		18 (34%)	32 (29%)	
New York	22 (42%)	24 (51%)		15 (28%)	29 (26%)	
Season of Cold			0.27			0.18
Fall (Sep-Nov)	15 (28%)	9 (19%)		14 (26%)	27 (24%)	
Winter (Dec-Feb)	19 (36%)	13 (28%)		25 (47%)	39 (35%)	
Spring (Mar-May)	14 (26%)	14 (30%)		8 (15%)	35 (31%)	
Summer (Jun-Aug)	5 (9.4%)	11 (23%)		6 (11%)	11 (9.8%)	
Race/Ethnicity			0.35			0.24
Black	23 (43%)	25 (53%)		33 (62%)	80 (71%)	
Hispanic	30 (57%)	22 (47%)		20 (38%)	32 (29%)	
BMI Percentile	86 (64, 98)	94 (70, 98)	0.31	82 (63, 98)	90 (73, 97)	0.59
Final Treatment Step Prescribed by Study Physician			0.16			0.44
1	2 (3.8%)	2 (4.3%)		8 (15%)	15 (13%)	
2	6 (11%)	7 (15%)		9 (17%)	22 (20%)	
3	2 (3.8%)	4 (8.5%)		5 (9.4%)	25 (22%)	
4	16 (30%)	18 (38%)		9 (17%)	14 (12%)	
5	12 (23%)	8 (17%)		14 (26%)	18 (16%)	
6	15 (28%)	8 (17%)		8 (15%)	18 (16%)	
Number of Positive Serum Specific IgE	3 (1, 3)	2 (1, 3)	0.21	2 (1, 3)	2 (1, 3)	0.83
Number of Positive Aeroallergen Skin Tests	6 (3, 7)	5 (4, 7)	0.75	5 (3, 7)	5 (3, 7)	0.70
Sensitized to at least one aeroallergen	53 (100%)	47 (100%)	>0.99	53 (100%)	112 (100%)	>0.99
Viral Cold Type						
RV	37 (70%)			22 (42%)		
EV	3 (5.7%)			1 (1.9%)		
Adenovirus	5 (9.4%)			4 (7.5%)		
Bocavirus	3 (5.7%)			12 (23%)		
Respiratory syncytial virus A/B	4 (7.5%)			3 (5.7%)		
Coronavirus	7 (13%)			10 (19%)		
Parainfluenza virus	2 (3.8%)			0 (0%)		
Metapneumovirus	1 (1.9%)			1 (1.9%)		
Influenza B	4 (7.5%)			3 (5.7%)		
Multiple Viruses	0 (0%)			0 (0%)		

[1] *Statistics presented: Median (IQR); n (%)*
Note: For subjects with 2 samples meeting specified criteria, both are included in the table.
[2] *Statistical tests performed: All p-values are from generalized linear mixed effect models with a random effect for participant ID to account for correlation between values from the same participant.*

Table S3 **ICATA cohort allergen sensitization data**

	Exacerbation Events			Non-exacerbation scheduled samples		
**Characteristic**	**Ex+Virus+**, N = 53[1]	**Ex+Virus-**, N = 47[1]	**p-value**[2]	**Ex-Virus+**, N = 53[1]	**Ex-Virus-**, N = 112[1]	**p-value**[2]
**# Positive Serum Specific IgE**	**3 (1, 3)**	**2 (1, 3)**	**0.21**	**2 (1, 3)**	**2 (1, 3)**	**0.83**
Sensitive to Alternaria (IgE)	22 (42%)	18 (38%)	0.77	27 (51%)	53 (47%)	0.66
Sensitive to Der p (IgE)	38 (72%)	26 (55%)	0.10	26 (49%)	54 (48%)	0.92
Sensitive to Der f (IgE)	35 (66%)	25 (53%)	0.21	25 (47%)	49 (44%)	0.68
Sensitive to German Cockroach (IgE)	31 (58%)	26 (55%)	0.82	19 (36%)	45 (40%)	0.59
**# Positive Aeroallergen Skin Tests**	**6 (3, 7)**	**5 (4, 7)**	**0.75**	**5 (3, 7)**	**5 (3, 7)**	**0.70**
Sensitive to Alternaria tenuis (Skin Test	19 (36%)	17 (36%)	>0.99	28 (53%)	58 (52%)	0.90
Sensitive to Aspergillus Mix (Skin Test)	14 (26%)	16 (34%)	0.43	17 (32%)	33 (29%)	0.73
Sensitive to Cat (Skin Test)	29 (55%)	29 (62%)	0.50	19 (36%)	55 (49%)	0.11
Sensitive to Cladosporium (Skin Test)	15 (28%)	10 (21%)	0.41	11 (21%)	26 (23%)	0.72
Sensitive to Der f (Skin Test)	20 (38%)	20 (43%)	0.64	19 (36%)	48 (43%)	0.39
Sensitive to Der p(Skin Test)	26 (49%)	23 (49%)	0.92	25 (47%)	60 (54%)	0.44
Sensitive to Dog (Skin Test)	16 (30%)	20 (43%)	0.21	17 (32%)	37 (33%)	0.90
Sensitive to German Roach (Skin Test)	35 (66%)	31 (66%)	0.91	31 (58%)	57 (51%)	0.36
Sensitive to MOUSE (Skin Test)	21 (40%)	17 (36%)	0.71	15 (28%)	37 (33%)	0.54
Sensitive to Penicillium (Skin Test)	12 (23%)	5 (11%)	0.12	13 (25%)	28 (25%)	0.95
Sensitive to Ragweed Mix (Skin Test)	15 (28%)	6 (13%)	0.068	23 (43%)	43 (38%)	0.54
Sensitive to Rat (Skin Test)	19 (36%)	17 (36%)	0.87	11 (21%)	31 (28%)	0.34
Sensitive to Roach Mix (Skin Test)	40 (75%)	31 (66%)	0.31	34 (64%)	67 (60%)	0.59
Sensitive to Timothy Grass (Skin Test)	16 (30%)	13 (28%)	0.74	18 (34%)	36 (32%)	0.82

[1] Statistics presented: Median (IQR); n (%)
Note: For subjects with 2 samples meeting specified criteria, both are included in the table.
[2] Statistical tests performed: All p-values are from generalized linear mixed effect models with a random effect for participant ID to account for correlation between values from the same participant.
